# Relationship Between Influenza, Temperature, and Type 1 Myocardial Infarction: An Ecological Time‐Series Study

**DOI:** 10.1161/JAHA.120.019608

**Published:** 2021-04-08

**Authors:** Alberto García‐Lledó, Sara Rodríguez‐Martín, Aurelio Tobías, Elvira García‐de‐Santiago, María Ordobás‐Gavín, Juan Carlos Ansede‐Cascudo, Joaquin Alonso‐Martín, Francisco J. de Abajo

**Affiliations:** ^1^ Department of Cardiology Hospital Universitario Príncipe de Asturias Alcalá de Henares Madrid Spain; ^2^ Department of Medicine University of Alcalá Alcalá de Henares Madrid Spain; ^3^ Código Infarto Madrid Servicio Madrileño de Salud Madrid Spain; ^4^ Clinical Pharmacology Unit Hospital Universitario Príncipe de Asturias Alcalá de Henares Madrid Spain; ^5^ Pharmacology Unit Department of Biomedical Sciences University of Alcalá Alcalá de Henares Madrid Spain; ^6^ Institute of Environmental Assessment and Water Research Spanish Council for Scientific Research Barcelona Spain; ^7^ School of Tropical Medicine and Global Health Nagasaki University Nagasaki Japan; ^8^ Epidemiology Department Directorate‐General of Public Health Madrid Regional Health Authority Madrid Spain; ^9^ Department of Cardiology Hospital Universitario de Getafe Madrid Spain

**Keywords:** cold, influenza, myocardial infarction, temperature, vaccine, Cardiovascular Disease, Epidemiology, Primary Prevention, Secondary Prevention, Risk Factors

## Abstract

**Background:**

Previous studies investigating the relationship of influenza with acute myocardial infarction (AMI) have not distinguished between AMI types 1 and 2. Influenza and cold temperature can explain the increased incidence of AMI during winter but, because they are closely related in temperate regions, their relative contribution is unknown.

**Methods and Results:**

The temporal relationship between incidence rates of AMI with demonstrated culprit plaque (type 1 AMI) from the regional primary angioplasty network and influenza, adjusted for ambient temperature, was studied in Madrid region (Spain) during 5 influenza seasons (from June 2013 to June 2018). A time‐series analysis with quasi‐Poisson regression models and distributed lag‐nonlinear models was used. The incidence rate of type 1 AMI according to influenza vaccination status was also explored. A total of 8240 cases of confirmed type 1 AMI were recorded. The overall risk ratio (RR) of type 1 AMI during epidemic periods, adjusted for year, month, and temperature, was 1.23 (95% CI, 1.03–1.47). An increase of weekly influenza rate of 50 cases per 100 000 inhabitants resulted in an RR for type 1 AMI of 1.16 (95% CI, 1.09–1.23) during the same week, disappearing 1 week after. When adjusted for influenza, a decrease of 1ºC in the minimum temperature resulted in an increase of 2.5% type 1 AMI. Influenza vaccination was associated with a decreased risk of type 1 AMI in subjects aged 60 to 64 years (RR, 0.58; 95% CI, 0.47–0.71) and ≥65 years (RR, 0.53; 95% CI, 0.49–0.57).

**Conclusions:**

Influenza and cold temperature were both independently associated with an increased risk of type 1 AMI, whereas vaccination was associated with a reduced risk among older patients.

**Nonstandard Abbreviation and Acronym**
IR: incidence rate

**Clinical Perspective**

**What Is New?**

- For the first time, this ecological study shows a relationship between influenza‐like illness and type 1 acute myocardial infarction, selected from a regional primary angioplasty database during 5 influenza seasons.
- We have found an independent relationship between type 1 acute myocardial infarction and influenza, and a relationship was also found with cold temperature.
- Influenza vaccination was associated with a reduced risk of type 1 acute myocardial infarction among patients aged >59 years; our results suggest that influenza viruses play a major role on plaque rupture.

**What Are the Clinical Implications?**

- The ST‐segment–elevation myocardial infarction networks should be reinforced during both cold weather and influenza seasons.
- Cold‐wave alerts should be considered even in regions with temperate climate.
- An effort must be done to reach the vaccination targets suggested by medical societies and Public Health Authorities; cardiologists have to actively participate in vaccine counseling as a primary and secondary prevention tool for ischemic heart disease.

Coronary heart disease, particularly acute coronary syndrome (ACS), is the leading cause of death and disability in the world,^1^ and influenza is one of the most common infections, with high morbidity and mortality.^2^ A relationship between both diseases has been suspected for some time.^3^ Several systematic reviews and meta‐analyses have suggested a significant association between respiratory infection and ACS.^4^, ^5^ More recently, it has been shown that serologically confirmed influenza virus infection was associated with an increased risk of acute myocardial infarction (AMI), strongly supporting this relationship.^6^, ^7^ It has been postulated that influenza could trigger an AMI, favoring the rupture of the atheroma plaque,^8^ but, as far as we know, no study has distinguished between type 1 infarction, induced by rupture of the plaque, and type 2 infarction, attributable to an imbalance in the oxygen supply.^9^ Any respiratory infection can cause tachycardia, hypoxia, and a systemic inflammatory response, which could contribute to myocardial necrosis by mechanisms other than plaque rupture.^10^, ^11^

Influenza and low temperatures are closely related in temperate zones.^12^ Although some authors attribute excess winter mortality and AMI risk to low temperatures,^12^, ^13^, ^14^ others consider that it is mainly attributable to the influenza epidemic.^15^ The independent contribution of each factor needs to be explored in the same study.

The main objective of our study was to analyze the temporal relationship between the incidence of influenza and episodes of ST‐segment–elevation myocardial infarction (STEMI), in which a culprit plaque had been demonstrated by angiography (type 1 AMI), adjusting for ambient minimum temperature. Also, we explored the effect of influenza vaccination on the population risk of type 1 AMI at community level.

## Methods

### Population and Registry “*Código Infarto Madrid*”

The data that support the findings of this study are available from the corresponding author on reasonable request.

The study population consists of STEMI cases from the “*Código Infarto Madrid*” registry from the Autonomous Community of Madrid (hereinafter, Madrid),^16^ in whom the existence of a culprit plaque was confirmed during cardiac catheterization. Data were collected between June 2013 and June 2018, date of the last audit. “*Código Infarto Madrid*” is a public program designed to coordinate the reperfusion treatment of STEMI in Madrid, which has been described in detail elsewhere.^14^ The researchers received an anonymized version of the official audited registry, including age, sex, date, and time of onset of symptoms, catheterization data (if done), and catheterization laboratory findings.

The STEMI alert implies the activation of a system, but not a definitive diagnosis. To eliminate those cases without a final diagnosis of STEMI, and to exclude other types of AMI than type 1, we included only those in which a culprit plaque was identified during the catheterization. The Research Ethics Committee of the University Hospital *Príncipe de Asturias*, one of the hospitals included in the registry, granted the approval for the study, including the exemption to request informed consent, given that the data were collected from anonymized records.

### Meteorological Data

The weekly averaged minimum temperature was calculated using the minimum daily temperature collected from a single observatory in Madrid (Getafe), which is considered the most representative of region temperatures.^14^ Minimum temperature was selected because previous studies^17^ reported that it is more closely related with AMI incidence than mean daily temperature. Data were obtained from the Spanish Meteorological Agency.^18^

### Influenza Data: Incidence Rates and Vaccination Status

The weekly incidence of influenza for each season throughout the study period, stratified by age groups (15–64 and ≥65 years) and sex, was obtained from the Epidemiological Surveillance Network in the region of Madrid. These data are estimated from 2 sources: (1) the Sentinel Physicians Network and (2) the Surveillance System for Notifiable Diseases.^19^, ^20^ The incidence estimated by this method reflects influenza‐like illness, not virologically confirmed influenza.

(1) The Sentinel Physicians Network is based on the voluntary participation of a sample of randomly selected primary care physicians. The population served by these primary care physicians constitutes a representative sample of Madrid population, according to demographic, economic, and cultural variables, and includes around 2% to 3% of Madrid population.^20^ The definition of a case of influenza follows that adopted in the European Union^21^: sudden appearance (<12 hours) of at least one general symptom (fever, malaise, headache, and/or myalgia) and at least one respiratory symptom (cough, sore throat, and/or dyspnea) in the absence of other diagnostic suspicion. The primary care physician is responsible for collecting pharyngeal samples from the first 2 suspected cases of influenza aged <60 years and treated in his/her practice every week and from all cases aged ≥60 years. The samples are sent to reference laboratories for reverse transcription–polymerase chain reaction detection of virus, isolation in Madin‐Darby canine kidney cells, and genetic and antigenic characterization. Data are sent weekly to the Institute of Health Carlos III, which is integrated into the European Group for Influenza Surveillance. The Sentinel Physicians Network monitors the incidence of influenza during the season (from week 40 to week 20 next year, roughly, from October to May). The period between 21st and 39th weeks is defined as noninfluenza period. The incidence is calculated weekly, using as numerator the number of reported cases (influenza‐like illness) and as denominator the population assigned to each sentinel primary care physician, adjusted for the number of working days providing medical care [adjusted population=(population×number of consultation days per week)/5].^19^

(2) The Surveillance System for Notifiable Diseases is integrated into the National Epidemiological Surveillance Network.^22^ All physicians are required to report cases of influenza diagnosed by the clinic or virologically confirmed. These cases are recorded in the Surveillance System for Notifiable Diseases registry. It uses the data from all the primary care centers of the Madrid Health Service, hospitals (public and private), and other institutions (private health, nursing homes, and correctional and military institutions). Since 2009, an automatic compilation of influenza cases has been performed in the electronic primary care medical record. Data from the Sentinel Physicians Network and the Surveillance System for Notifiable Diseases are combined to obtain an estimate of the incidence of influenza, which is in fact an "influenza‐like illness" estimation.

During an influenza season, we took as epidemic periods those officially designed as such by the Directorate‐General of Public Health. The epidemic period is declared when the weekly incidence rate (IR) exceeded an "epidemic threshold," determined by the upper limit of the 95% CI of the average of the 30 pre‐epidemic weekly IRs from the past 10 seasons (excluding the pandemic one, 2009–2010).^20^

The vaccination status of the patients who experienced a type 1 AMI was provided by the Public Health Service of Madrid after crossing the *Código Infarto Madrid* registry with the influenza vaccination database, which records all doses of influenza vaccine administered through the Madrid Health Service system, including nursing homes. A patient was considered effectively vaccinated if he/she received the vaccine beyond 14 days before the ischemic event.^23^ Those receiving the vaccine after the AMI were included among nonvaccinated, and those receiving it within the time window of 14 days before the AMI were excluded from the main analysis.

### Statistical Analysis

The IRs of type 1 AMI in the population aged ≥15 years of Madrid were computed using as numerator the number of valid cases per day from the *Código Infarto Madrid* registry and as denominator the population aged ≥15 years from Madrid, provided by the official census of corresponding calendar year (measured on January 1).^24^ The IR was expressed per 100 000 person‐weeks.

The association between the weekly IRs of influenza and type 1 AMI in Madrid was evaluated using an ecological time‐series design.^25^ Data were analyzed with quasi‐Poisson regression models. The association of influenza with type 1 AMI may not be immediate and/or may persist for a time; for this reason, we evaluated possible delayed effects up to 2 weeks after the week in which the case was reported. Thus, we used a 2‐week distributed lag model.^26^ We evaluated the linearity of the associations without observing evidence of departure from linearity. Likewise, the association was also studied by sex (male and female) and age groups (<65 and ≥65 years). In all regression models, confounding by time trend and seasonality was controlled for using indicator variables for calendar time (ie, year, month, and week). We also controlled for the averaged minimum weekly temperature and influenza epidemic week.

The impact of influenza vaccine on the incidence of type 1 AMI was explored using the data from the *Código Infarto Madrid* registry, the status of vaccination of AMI cases, and the population statistics of influenza vaccination coverage in the region provided by the Public Health Service of Madrid, all of them during every influenza season. The incidence of type 1 AMI among vaccinated was estimated using the number of vaccinated AMI cases as the numerator and the official statistics on population vaccinated provided by the Public Health Service as the denominator. Type 1 AMI cases vaccinated after the event and during the 14 days before the event were excluded from both numerator and denominator. The incidence of type 1 AMI among nonvaccinated in every epidemic season was estimated using the number of nonvaccinated AMI cases as the numerator (including those vaccinated after the event) and the population census of Madrid from which the vaccinated population was subtracted. To this figure, we added the number of people receiving the vaccine after the AMI. The incidence was estimated by 3 age groups (14–59, 60–64, and ≥65 years, as provided by the official influenza database). Similarly, we estimated the incidence of AMI among nonvaccinated per each age group and then we calculated the risk ratio (RR) and the 95% CI overall, per each age group, and per season. In a secondary analysis, we estimated the type 1 AMI incidence by vaccination status during noninfluenza periods (from 21st to 39th week).

As a sensitivity analysis, we performed a correction for a potential adherent‐user bias. To this end, we assumed that the RR of vaccinated versus nonvaccinated during nonflu seasons is entirely attributable to an adherence bias (the worst‐case scenario). Then, this effect was discounted from the observed effect of vaccination during flu seasons to estimate the "true" RR attributed to a biological effect of vaccination on type 1 AMI (RR attributed to a biological effect of vaccination=RR observed/adherence bias; see Data S1).

All the statistical analyses were performed using the Stata statistical software, release 16 (StataCorp, College Station, TX; 2019).

## Results

Over the 5 seasons of the study period, the registry recorded 8240 cases of type 1 AMI. Of them, 5553 (67.6%) occurred during influenza seasons, 4232 (76.2%) were men, and 2536 were aged ≥65 years (45.7%). The IRs for the whole study period were 0.33, 1.13, and 1.34 per 100 000 person‐weeks for the age groups of 15 to 59, 60 to 64, and ≥65 years, respectively. The main characteristics of type 1 AMI cases and specific IRs are shown in Table 1. IRs were consistent across different seasons (shown in Table S1).

**Table 1:** Main Characteristics of Type 1 AMI Cases and Specific IRs

Characteristics	Influenza Seasons (40th–20th Week) (N=5553)		Noninfluenza Periods (21st–39th Week) (N=2687)		Overall (N=8240)	
	Cases, N (%)	IR	Cases, N (%)	IR	Cases, N (%)	IR
Sex						
Men	4232 (76.2)	1.02	2065 (76.9)	0.80	6297 (76.4)	0.94
Women	1321 (23.8)	0.29	622 (23.2)	0.22	1943 (23.6)	0.26
Age group, y						
15–59	2353 (42.4)	0.36	1160 (43.2)	0.28	3513 (42.6)	0.33
60–64	664 (12.0)	1.24	320 (11.9)	0.95	984 (12.0)	1.13
≥65	2536 (45.7)	1.47	1207 (44.9)	1.12	3743 (45.4)	1.34
Coronary artery						
Territory	2271 (40.9)	…	1074 (40.0)	…	3345 (40.6)	…
Anterior	2505 (45.1)	…	1251 (45.2)	…	3720 (45.2)	…
Other site not defined	777 (14.0)	…	398 (14.8)	…	1175 (14.3)	…
Season						
2013–2014	1404 (25.3)	0.80	626 (23.3)	0.57	2030 (24.6)	0.71
2014–2015	931 (16.8)	0.53	591 (22.0)	0.54	1522 (18.5)	0.54
2015–2016	1015 (18.3)	0.58	408 (15.2)	0.38	1423 (17.3)	0.50
2016–2017	910 (16.4)	0.52	540 (20.1)	0.50	1450 (17.6)	0.51
2017–2018	1293 (23.3)	0.74	522 (19.4)	0.48	1815 (22.0)	0.64
Whole study period	5553 (100)	0.64	2687 (100)	0.49	8240	0.58

AMI indicates acute myocardial infarction; and IR, incidence rate (per 100 000 person‐weeks).

### Influenza and Type 1 AMI Relationship: Time‐Series Analysis

**Figure 1:**
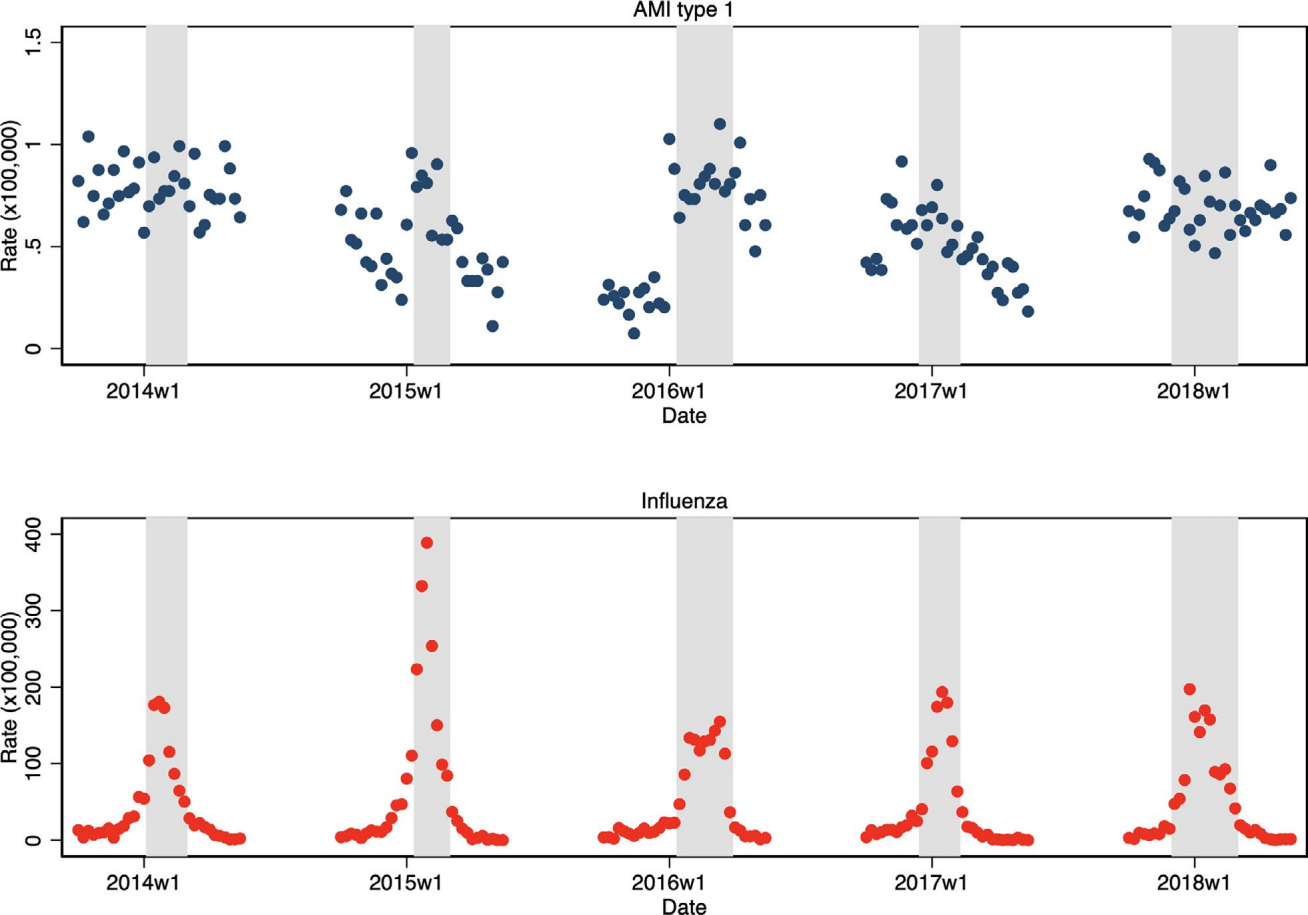
Scatterplot of the weekly incidence rates of type 1 acute myocardial infarction (AMI) (top) and influenza (bottom) during the influenza seasons (from 40th week to 20th week next year). In gray, it is shown the periods when the incidence rates of influenza exceeded the officially defined "epidemic threshold" per every influenza season ("epidemic periods"). W1 indicates week 1.

The weekly IRs of type 1 AMI and influenza per every season are shown in Figure 1. The overall IR of type 1 AMI during the epidemic periods of influenza was 0.73 per 100 000 person‐weeks (95% CI, 0.69–0.77) compared with 0.57 (95% CI, 0.53–0.61) during the nonepidemic periods, yielding to a crude RR of 1.28 (95% CI, 1.15–1.44) and an adjusted RR of 1.23 (95% CI, 1.03–1.47) (adjusted for year, month, and average weekly minimum temperature). The increased risk found was consistent by sex and age groups, although in men and younger subjects, the adjusted RR was marginally nonsignificant (Table 2). After adjusting for influenza, minimum temperature showed an independent effect on type 1 AMI, whose risk increased linearly by 2.5% per 1°C decrease of the minimum temperature.

**Table 2:** IRs of Type 1 AMI (IR Expressed per 100 000 Person‐Weeks) and the Corresponding RRs During Epidemic Compared With Nonepidemic Periods of All Influenza Seasons Combined, in the Total Population and in Different Subgroups

Variable	Nonepidemic Periods of Influenza Seasons		Epidemic Periods of Influenza Seasons		Crude		Adjusted*	
	IR	95% CI	IR	95% CI	RR	95% CI	RR	95% CI
Total	0.57	0.53–0.61	0.73	0.69–0.77	1.28^†^	1.15–1.44	1.23	1.03–1.47
Sex								
Women	0.25	0.23–0.27	0.34	0.32–0.37	1.37^†^	1.19–1.57	1.35^†^	1.05–1.73
Men	0.92	0.85–0.99	1.16	1.09–1.24	1.26^†^	1.12–1.42	1.19	0.99–1.43
Age, y								
15–64	0.39	0.36–0.42	0.49	0.45–0.53	1.26^†^	1.12–1.42	1.22	1.01–1.48
≥65	1.28	1.18–1.40	1.67	1.57–1.77	1.30^†^	1.14–1.48	1.25^†^	1.01–1.55

AMI indicates acute myocardial infarction; IR, incidence rate; and RR, risk ratio.

* Adjusted for year, month, and weekly average of minimum temperature.

^†^ Xxxx.

**Figure 2:**
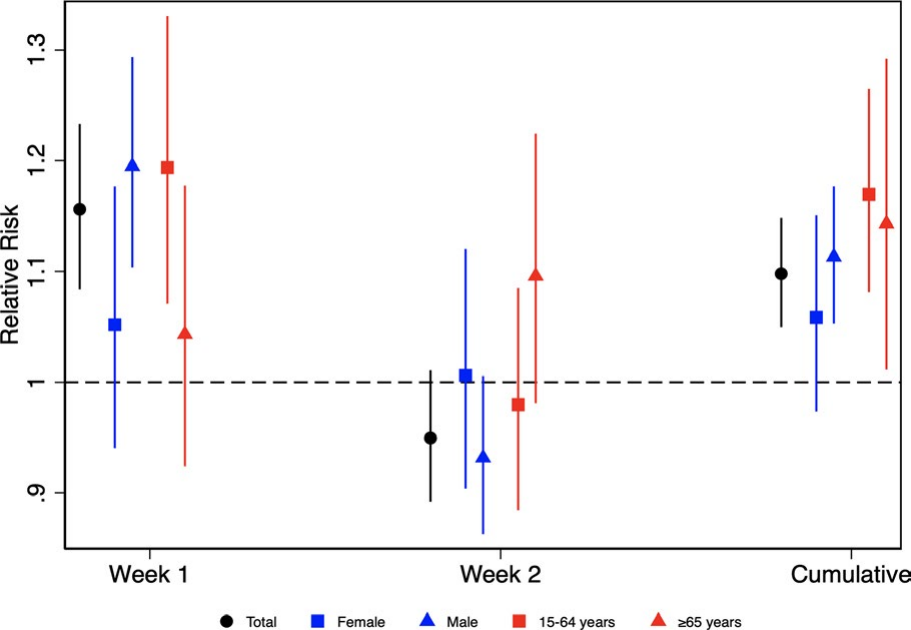
Results of time‐series analysis using a distributed lag nonlinear model. For the whole sample, relative risk of type 1 acute myocardial infarction, estimated during the same week of flu infection (week 1), the next week (week 2), and when both are joined (cumulative), is shown.

In the distributed lag nonlinear model analysis, an increase in the weekly influenza rate of 50 cases per 100 000 inhabitants resulted in an increased risk of AMI during the same week when influenza cases were reported (RR, 1.16; 95% CI, 1.09–1.24), disappearing in the next week (RR, 0.95; 95% CI, 0.89–1.05) (Figure 2). The association did not reach statistical significance in women and older people. When the cumulative effect of the 2 weeks was analyzed, the association was significant overall (RR, 1.1; 95% CI, 1.05–1.15) and in both age groups (RR_15–64 years_, 1.17 [95% CI, 1.08–1.27]; and RR_≥65 years_, 1.15 [95% CI, 1.02–1.30]) (Figure 2). In women, the cumulative RR was marginally nonsignificant (RR, 1.06; 95% CI, 0.98–1.15) (Figure 2 and Table S2).

### Effect of Influenza Vaccination

**Figure 3:**
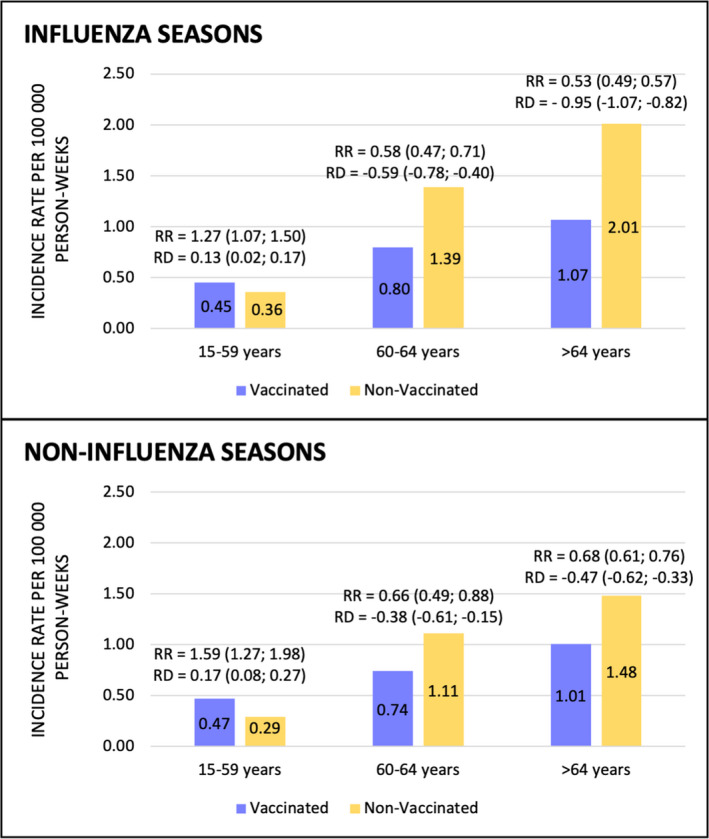
Incidence rates of type 1 acute myocardial infarction among vaccinated and nonvaccinated population by different age groups during influenza season (A) and noninfluenza period (B). RD indicates rate difference between vaccinated and unvaccinated; and RR, relative risk of vaccinated compared with unvaccinated.

Of 5553 type 1 AMI cases occurring during the 5 influenza seasons, 1299 (23.4%) had a record of vaccination at least 15 days before the ischemic event, and 4161 (74.9%) had no such record. In 93 cases (1.7%), there was a record of influenza vaccination within the time window of 14 days before the event, and these cases were excluded from the main analysis. The IRs of type 1 AMI among vaccinated and nonvaccinated populations in Madrid over the influenza seasons by age group are shown in Figure 3A. The resulting overall RRs in vaccinated versus nonvaccinated people among those aged ≥65 and 60 to 64 years were 0.53 (95% CI, 0.49–0.57) and 0.58 (95% CI, 0.47–0.71), respectively, whereas it was 1.27 (95% CI, 1.07–1.50) among subjects aged <60 years. The results were consistent across the 5 seasons analyzed (Figure S1 and Table S3).

Of 2687 type 1 AMI cases occurring during nonflu seasons (from 21st to 39th weeks), 735 (27.4%) had a record of past influenza vaccination, whereas 1952 (72.6%) had no such a record. The respective IRs by age group are shown in Figure 3B. A reduced risk was also observed in vaccinated compared with nonvaccinated subjects among 60 to 64 years (RR, 0.66; 95% CI, 0.49–0.88) and ≥65 years groups (RR, 0.68; 95% CI, 0.61–0.76). Interestingly, there was barely any difference in the IRs of type 1 AMI between influenza season and no‐influenza period in vaccinated population, whereas among nonvaccinated subjects, the IRs were higher during influenza season compared with noninfluenza period in older age groups: for those aged 60 to 64 years, IR of 1.39 versus 1.11 per 100 000 person‐weeks (*P*<0.01 for the difference); and for those aged ≥65 years, IR of 2.01 versus 1.48 per 100 000 person‐weeks (*P*=0.001 for the difference).

### Sensitivity Analysis

Assuming the risk reduction observed during noninfluenza period as a measure of an adherent‐user bias, the “true” RRs would be 0.88 (95% CI, 0.71–1.08) and 0.78 (95% CI, 0.72–0.84) for age groups of 60 to 64 and ≥65 years, respectively.

## Discussion

This study analyzed a large series of type 1 AMI cases attended for 5 years by a public healthcare network in a single region within a temperate zone, which experiences both wide thermal variations^14^, ^18^ and seasonal peaks of epidemic influenza.^19^ Our results show an independent relationship between influenza‐like illness and type 1 AMI, adjusted for ambient temperature. Cold temperature has also been shown to be an independent factor associated with type 1 AMI risk. A lower incidence of type 1 AMI was observed among vaccinated people aged ≥60 years during each influenza season.

The relationship between epidemic periods of influenza and the increase in mortality from other causes has been described^3^ and is included in the concept of "excess winter mortality" observed in temperate zones.^27^, ^28^ Part of the excess mortality is attributable to an increased incidence of ACS, reported by numerous studies.^12^, ^13^, ^14^, ^15^ Both cold temperatures and influenza infection are postulated to cause this increased risk of death and ACS, although the relative contribution of each factor is a matter of controversy, because low temperatures and influenza are closely associated in temperate regions.^15^, ^27^, ^28^, ^29^, ^30^, ^31^ The natural experiment that happened during the fall pandemic of 2009 suggests an independent relationship between influenza, AMI, and temperature.^32^ Although an increased risk of ACS has also been reported with other respiratory and nonrespiratory infections,^3^, ^8^, ^10^, ^12^, ^33^ the relationship with influenza is well studied.^3^, ^4^, ^5^, ^6^, ^7^, ^10^, ^34^

Influenza could be associated with the development of coronary atherosclerosis, a greater vulnerability of atheroma plaques, and an inflammatory and prothrombotic state that would favor type 1 myocardial infarction.^8^, ^9^, ^10^, ^33^ However, in situations of systemic stress and hypoxemia, as happens during severe respiratory infections, an increased risk of type 2 infarction is also possible, clinically displayed as non–ST‐segment–elevation myocardial infarction (NSTEMI). To date, few studies have considered separately the relationship between influenza and different types of AMI, although one study described an important relative increase in the rate of NSTEMI versus STEMI during influenza season (9:1) compared with noninfluenza period (3:1).^35^ Also, up to 2.9% of patients with influenza have been reported to present an elevated serum level of troponin, half of them without a final diagnosis of ACS.^11^ Myocarditis, sepsis, or impaired renal function can be accounted for a biomarker elevation, which may lead to an incorrect diagnosis of ACS, most frequently classified as NSTEMI.

To our knowledge, this study is the first specifically designed to assess the relationship of influenza‐like illness with type 1 AMI. The restriction to STEMI cases with a confirmed culprit lesion by angiography explains why our population is younger and with a higher proportion of men than those studies including also NSTEMI,^7^, ^11^, ^35^ as the latter is more common in women and older people.^11^ In accordance with previous studies,^3^, ^4^, ^5^, ^6^, ^7^, ^31^, ^34^ we found that epidemic peaks were temporally associated with an increased risk of AMI, but our study provides 2 important novelties: the association we found was necessarily with type 1 AMI (rupture of atheroma plaque) and the demonstration that both influenza‐like illness and cold temperature are independently associated with the risk of type 1 AMI. As in other studies,^6^, ^36^, ^37^ the greatest increased risk occurred in the same week influenza was reported. A recent study has shown that initial intrinsic defense against influenza is mediated by platelet‐neutrophil cross‐communication that regulates host response, but can also lead to thrombotic process activation.^38^ This could help explain this close association.

There is evidence that influenza vaccination is associated with a reduction in the risk of ischemic events in both primary^16^, ^18^, ^39^, ^40^ and secondary prevention.^27^, ^41^, ^42^ Even though this evidence is considered not definitive by some authors,^43^ mainly when used as primary prevention,^44^ influenza vaccine is included in most guidelines for the prevention of ischemic heart disease. In our study, the risk reduction associated with vaccination among patients aged ≥60 years was around 45% overall and shown to be consistent across the 5 seasons studied, which is in accordance with results found in other studies.^42^, ^45^ The lack of risk reduction of type 1 AMI among younger people is likely explained by a selection bias, as vaccination is mainly channeled in this age group to those who present established heart disease or are at high cardiovascular risk. Also, previous studies have shown that the attributable risk of AMI caused by influenza increases with age.^32^ This may help explain the greater effect of vaccines in protecting AMI in older people, even though the vaccine may cause a weaker response.^23^ An interesting finding of our study is that a risk reduction, albeit smaller, was also observed among vaccinated people during noninfluenza periods, also reported in other studies.^46^ It has been postulated that influenza vaccine can modulate the inflammatory status in patients with cardiovascular risk factors and this way could protect from atherothrombotic events beyond the prevention of influenza infection.^46^ It is also likely that an adherent‐user bias could partly account for such risk reduction among vaccinated subjects (as explained in limitations below). It is interesting to note that, as shown in Figure 3, among vaccinated people aged >60 years there was no difference in the IR of type 1 AMI between influenza season and noninfluenza period, whereas there were relevant differences between influenza and noninfluenza seasons among unvaccinated people aged >60 years. Altogether, these results support the recommendation to vaccinate all patients at risk of presenting an AMI. In our population, the vaccine coverage over the study period was around 56% to 60% among people aged ≥65 years, 25% to 30% among people aged 60 to 65 years, and around 4% to 6% in younger populations,^47^ figures that are higher than the ones observed in other regions of Spain and in other countries, but still far from the objectives set in both the United States and Europe.^27^, ^48^

### Limitations

First, the registry does not include all cases of STEMI occurring in the region, so the IRs are slightly underestimated. Also, it is possible that some STEMI cases could have received an influenza vaccine outside the public network and thus been misclassified among nonvaccinated. However, the coverage of the National Health System is 99.1% of the population,^47^ so the underrecording of both STEMI cases and vaccinations was most likely low and barely had an impact on our results. A portion of NSTEMI cases may be type 1 AMIs and are not included in STEMI network registries. Thus, our study does not include all type 1 AMIs occurring in our area, but all cases included in our study are type 1 AMIs. Second, this is an ecological study and has all the inherent limitations of this kind of study. In particular, we cannot know whether individual patients had influenza or not in the days before the STEMI. Third, it would have been of great interest to distinguish between vaccination in primary and secondary prevention, but unfortunately this information was not available; however, bearing in mind that the study was performed in the whole population, we may assume that vaccination was mostly done in a primary prevention scenario. Fourth, various strains of the influenza virus may have different effects on cardiovascular risk; also, differences between the circulating influenza viruses and those included in the available vaccines can result in low or suboptimal vaccine effectiveness during some seasons.^49^ The design of our study does not allow the detection of such strain‐specific effects. Fifth, as in all other observational studies of vaccination, it may be difficult to disentangle the biological effect attributable to the vaccine from the effect attributable to a better adherence to other prevention measures (eg, medications and modification in lifestyle factors, such as smoking, diet, and exercise) in vaccinated subjects.^50^ However, the correction done for a potential adherent‐user bias yielded risk reductions that are still clinically relevant and statistically significant among those aged ≥65 years. Sixth, other confounding factors, such as humidity or time indoor, were not considered in the present study. However, in a previous study,^14^ we did not find a relationship between humidity and STEMI rates (data not published), probably because Madrid has a dry continental climate with scarce variation in humidity along the year. Seventh, the age bands used in this study (14–59, 60–64, and ≥65 years) are the ones provided by health authorities, but narrower bands of <60 and >65 years would have been more appropriate. Eight, for legal reasons, the data on AMI cases are fully anonymized and, thus, it is possible that a given patient presents more than one episode; however, according to an estimated rate of AMI recurrence of 6% over 5 years, the estimated number of patients with >1 episode would be around 470 in our study; on the other hand, it is likely that AMI recurrences are associated with influenza in an independent manner each year, so it is possible to consider all episodes as independent events, including recurrences.

### Strengths

The main strengths of this study are as follows: (1) the type 1 AMI case definition validated by cardiac catheterization, which focuses the study on a specific pathophysiological process (plaque rupture), making false positives highly unlikely; (2) the large sample size and the inclusion of various epidemic periods; (3) the high and homogeneous coverage of the Spanish National Health System; and (4) the performance of a time‐series analysis that allowed us to control for trend and temperature.

## CONCLUSIONS

There is an independent relationship between the IR of seasonal influenza‐like illness and STEMI caused by plaque rupture (type 1 AMI), mainly during the same week of influenza reporting. Low temperatures are also independently associated with type 1 AMI. Both influenza and low temperature can explain the increase of type 1 AMI during cold periods in temperate areas, and part of the excess winter mortality. These facts must be considered when planning type 1 AMI assistance. Also, our data support the protective effect of influenza vaccine on cardiac atherothrombotic events, remembering the need of extending influenza vaccination campaigns to the whole vulnerable population.

## Sources of Funding

This work has been partially funded by unrestricted research grants from the *Fundación para la Investigación Biomédica*, University Hospital “*Príncipe de Asturias*” (*Servicio Madrileño de Salud*, Madrid, Spain) (to Drs García‐Lledó and de Abajo) and funding from Sanofi‐Pasteur S.A., a manufacturer of influenza vaccines (to Dr de Abajo). The funders had no role in the study design, data collection, data analysis, data interpretation, or writing the report. Dr Tobías received support from the Japanese Society for the Promotion of Science in its invitation‐to‐research fellowship program in Japan (S18149).

## Disclosures

Dr de Abajo reports grants from Sanofi‐Pasteur, from null, during the conduct of the study.

## Supporting information


Data S1. Correction for Adherent Bias
Tables S1–S3
Figure S1
