# Supplementary material for: Relationship Between Influenza, Temperature, and Type 1 Myocardial Infarction: An Ecological Time‐Series Study
Source: J Am Heart Assoc. 2021 Apr 8;10(8):e019608. doi: 10.1161/JAHA.120.019608 (PMC8174174; doi:10.1161/JAHA.120.019608)

SUPPLEMENTAL MATERIAL

Data S1.

Correction for Adherent Bias

This correction assumes that the risk reduction of type-1 AMI observed among vaccinated population during non-influenza seasons is entirely attributed to adherence to other preventive measures (life-style factors and medications) and that this effect on AMI is constant within each age subgroup. Under this assumption, vaccination in non-influenza seasons will be acting merely as a marker of adherence to treatment with no specific biological effect. According to this, the RR of vaccinated vs. non-vaccinated in non-influenza seasons could be used as a measure of the adherence bias (RR_{bias}). Then, this part could be discounted from the observed effect (RR_{crude}) of vaccination during influenza seasons to obtain the "true" RR (RR_{true}) attributed to a biological effect of vaccination on type-1 AMI. The following example is for the subgroup aged 65 years or older, but the same logic applies to other subgroups:

RR_{crude} = Observed RR during influenza seasons among 65 years or older

$= IR_{11} / IR_{10} = 0.53$

Where,

IR_{11} =Incidence rate during influenza seasons among vaccinated

IR_{10} = Incidence rate during influenza seasons among non-vaccinated

RR_{bias} =Observed RR during non-influenza seasons among 65 years or older (adherence bias)

$= IR_{01} / IR_{00} = 0.68$

Where,

IR_{01} =Incidence rate during non-influenza seasons among vaccinated

IR_{00} = Incidence rate during non-influenza seasons among non-vaccinated

Then, if the non-vaccinated population during the influenza seasons had the same adherence to preventive measures as the vaccinated population, the IR among them once corrected for adherence would be:

Corrected IR_{10} = IR_{10} * RR_{bias}

Then,

RR_{true} = IR_{11} / Corrected IR_{10}

= IR_{11} / IR_{10} * RR_{bias}

= $RR_{observed}$ / RR_{bias}

= 0.53 / 0.68 = 0.78

Table S1. Incidence rates of type-1 AMI (expressed per 100,000 person-season) in the total population and in different age subgroups by flu season.

Flu season	Overall	15-59 years	60-64 years	≥65 years
2013-2014	25.62	14.48	49.13	62.85
2014-2015	17.11	9.46	30.02	42.54
2015-2016	18.71	10.49	39.16	43.06
2016-2017	16.69	10.15	30.59	36.27
2017-2018	23.56	13.54	48.86	51.49
Whole study period	20.35	11.63	39.63	47.09

Table S2. Relative risks of type-1 AMI estimated during the same week of influenza infection (week 1), the next week (week 2), and when both weeks are joined (cumulative), overall and by sex and age groups.

	Relative Risk (95%CI)		
	**Week 1**	**Week 2**	**Cumulative**
**Total**	1.16 (1.09-1.24)	0.95 (0.89-1.01)	1.10 (1.05-1.15)
**Sex**			
Female	1.06 (0.95-1.18)	1.00 (0.90-1.12)	1.06 (0.98-1.15)
Male	1.20 (1.11-1.30)	0.93 (0.86-1.00)	1.12 (1.05-1.18)
**Age**			
15-64	1.20 (1.08-1.34)	0.98 (0.88-1.08)	1.17 (1.08-1.27)
≥65	1.06 (0.94-1.20)	1.09 (0.97-1.21)	1.15 (1.02-1.30)

Table S3. Relative risk of type-1 AMI in vaccinated vs. non-vaccinated persons during each influenza season by age group.

	**Relative Risk (95%CI)**		
	**Age group in years**		
**Season**	**15-59**	**60-64**	**≥65**
2013-2014	1.24 (0.84-1.76)	0.51 (0.32-0.78)	0.51 (0.43-0.60)
2014-2015	1.05 (0.63-1.67)	0.71 (0.40-1.17)	0.51 (0.42-0.62)
2015-2016	1.71 (1.18-2.41)	0.59 (0.36-0.94)	0.62 (0.51-0.75)
2016-2017	1.40 (0.92-2.04)	0.55 (0.31-0.94)	0.52 (0.42-0.65)
2017-2018	1.05 (1.07-1.50)	0.57 (0.37-0.86)	0.49 (0.42-0.59)
Whole study period	1.27 (1.07-1.50)	0.58 (0.47-0.71)	0.53 (0.49-0.57)

Figure S1. Incidence rates of type-1 AMI per 100,000 persons among vaccinated and non-vaccinated population, by age group and influenza season.

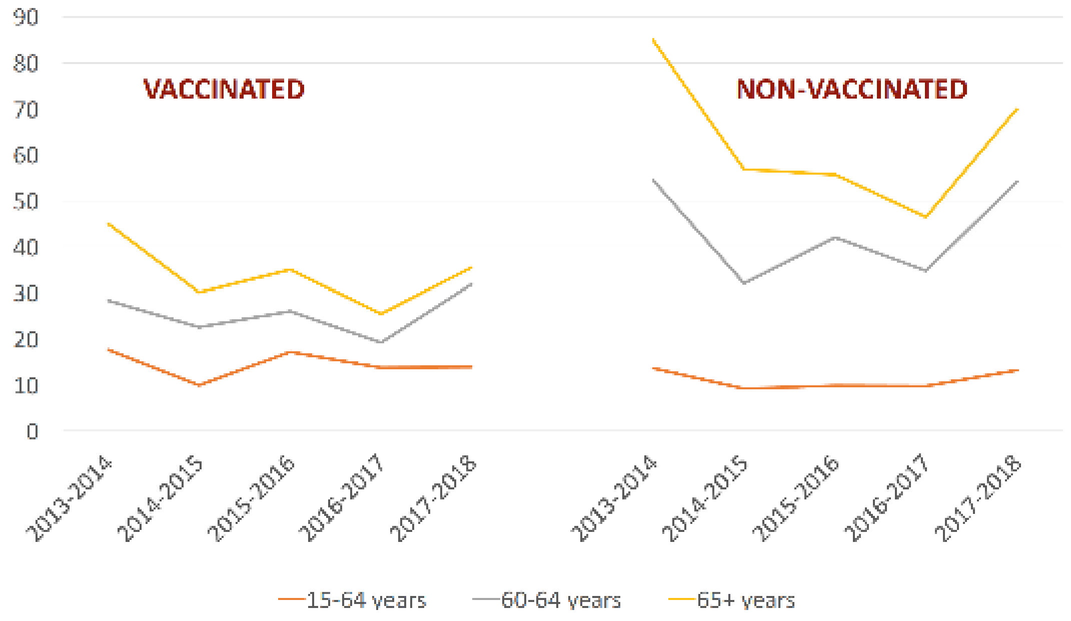

Supplement: Supplementary file 1 — Data S1. Correction for Adherent Bias Tables S1–S3 Figure S1 [file JAH3-10-e019608-s001.pdf]
